# Parkin plays a crucial role in acute viral myocarditis by regulating mitophagy activity

**DOI:** 10.7150/thno.97675

**Published:** 2024-08-26

**Authors:** Yixuan Qiu, Jing Xu, Yilian Chen, Yihao Wu, Yuan-nan Lin, Weike Liu, Zhening Wang, Yuqing Wu, Xinge Qian, Yue-Chun Li

**Affiliations:** From the Department of Cardiology, Second Affiliated Hospital and Yuying Children's Hospital of Wenzhou Medical University, Wenzhou, China.

**Keywords:** viral myocarditis, mitophagy, NF-κB pathway, Parkin, inflammation

## Abstract

**Rationale:** Parkin (an E3 ubiquitin protein ligase) is an important regulator of mitophagy. However, the role of Parkin in viral myocarditis (VMC) remains unclear.

**Methods:** Coxsackievirus B3 (CVB3) infection was induced in mice to create VMC. Cardiac function and inflammatory response were evaluated by echocardiography, histological assessment, and molecular analyses. AAV9 (adeno-associated virus 9), transmission electron microscopy (TEM) and western blotting were used to investigate the mechanisms by which Parkin regulates mitophagy and cardiac inflammation.

**Results:** Our data indicated that Parkin- and BNIP3 (BCL2 interacting protein 3 like)-mediated mitophagy was activated in VMC mice and neonatal rat cardiac myocytes (NRCMs) infected with CVB3, which blocked autophagic flux by inhibiting autophagosome-lysosome fusion. Parkin silencing aggravated mortality and accelerated the development of cardiac dysfunction in CVB3-treated mice. While silencing of Parkin did not significantly increase inflammatory response through activating NF-κB pathway and production of inflammatory cytokines post-VMC, the mitophagy activity were reduced, which stimulated the accumulation of damaged mitochondria. Moreover, Parkin silencing exacerbated VMC-induced apoptosis. We consistently found that Parkin knockdown disrupted mitophagy activity and inflammatory response in NRCMs.

**Conclusion:** This study elucidated the important role of Parkin in maintaining cardiac function and inflammatory response by regulating mitophagy activity and the NF-κB pathway during acute VMC. Although the functional impact of mitophagy remains unclear, our findings suggest that Parkin silencing may accelerate VMC development.

## Introduction

Viral myocarditis (VMC) remains a leading cause of heart failure and sudden cardiac death in young adults and is characterized by inflammatory cell infiltration 1, 2. VMC is not only characterized by cardiomyocyte death, but also by the parallel activation of innate and adaptive immune responses and the generation of inflammatory factors. As key drivers, cardiomyocytes are involved in the occurrence and regulation of cardiac inflammation 3, 4. Accumulating evidence has demonstrated that mitochondrial damage has harmful consequences that contribute to the progression of myocarditis and dilated cardiomyopathy 5. Despite some advances, specific pharmacological treatments for VMC are not currently available. Therefore, new therapeutic strategies are urgently required to prevent VMC.

Mitophagy, a selective autophagic process, removes excess or damaged mitochondria to maintain a healthy mitochondrial population, and is involved in numerous physiological and pathological processes 6. The PINK1/Parkin typical mitophagy pathway is one of the most studied pathways for mitochondrial clearance. PINK1 (PTEN)-induced putative kinase 1, which targets the mitochondria, stabilizes and accumulates on the outer mitochondrial membrane when the mitochondrial membrane potential is lost. PINK1 then promotes the recruitment of Parkin, an E3-ubiquitin ligase that ubiquitinates a number of mitochondrial proteins on the outer mitochondrial membrane of damaged mitochondria and is vital for removing defective mitochondria 7. Previous studies have shown that CVB3 infection disrupts mitochondrial dynamics and induces mitophagy in neural progenitor cell, HeLa and H9C2 cardiomyocytes, suppressing the activation of interferon signaling to enhance viral replication 8, 9. However, mitophagy and the alteration of autophagic flux in animal models during VMC and its precise role in the pathophysiology of VMC remain poorly understood.

Thus, the present study sought to elucidate the role of mitophagy activation during VMC, utilizing AAV9 (adeno-associated virus 9)-mediated Parkin silencing mice and cultured NRCMs with CVB3 infection as a model of VMC in the heart. The goals of this study were to (i) demonstrate the activation of mitophagy during VMC as well as to investigate the underlying mechanisms involved, and (ii) clarify the significance of Parkin-mediated mitophagy in cardiac inflammation and cardiac function in the presence of acute VMC.

## Methods

### Animals

4-week-old male BALB/c mice were obtained from Zhejiang Vital River Experimental Animal Technology Co. Ltd. All the animal procedures were performed in accordance with the Guide for the Care and Use of Laboratory Animals published by the National Institutes of Health and were approved by the Institutional Animal Ethics Committee of Wenzhou Medical University.

### Construction of adeno-associated virus carrying a short hairpin-Parkin plasmid

A U6-MCS-CAG-EGFP vector carrying the adenovirus9-shRNA-Parkin (a short hairpin RNA for Parkin, that can be used to suppress Parkin gene expression) plasmid (AAV9-shParkin) and negative controls (AAV9-shNC) were generated by a company (Genechem Co Ltd, Shanghai, China). For in vivo infection, AAV9 particles were injected into the mice via the tail vein at a dose of approximately 2 x 10^11^ vector genome copies (vg)/mice two weeks before inducing VMC, according to a previously described protocol 10. The infection efficiency was assessed by fluorescence microscopy with enhanced green fluorescent protein (EGFP) fluorescence (510 nm) in frozen slices. The mice were subjected to echocardiography, and cardiac tissues were obtained for western blotting and histological analyses. Please refer to the Supplementary Material for more details.

### Western blotting

Total protein was extracted from mouse hearts and cultured NRCMs, which was performed as described previously 11. Primary antibodies include LC3A/B, p62, Caspase-3, GAPDH, Caspase-3/p17/p19, Bax, Parkin, RELA/NF-κB p65, BNIP3, VP1 and Phospho-NF-κB p65 (Ser536). The protein abundance was evaluated by immunoblotting with at least three biological replicates. Please refer to the Supplementary Material for more details.

### SiRNA transfection

Neonatal SD rat pups (0-3 days old) were purchased from Charles River and sacrificed for cardiomyocyte extraction. SiRNA knockdown was performed as described previously 12, 13. NRCMs were seeded in a 6-well dish at 8 × 10^4^/well and were transfected with a mixture containing 50 nmol Parkin siRNA (RiboBio, China), 6μL lipofectamine 2000 (Invitrogen, USA) and 244μL Opti-MEM (Gibco, USA). Target sequences are listed in the Supplementary Material (Table S1). Non-targeting scrambled siRNAs were used as negative controls (RiboBio, China). Experiments were performed after an additional 24 h.

### Echocardiography

Echocardiograms were performed on mice anesthetized with 1.5% isoflurane (Vevo 2100 system, MS400C probe, Canada). The mice were anaesthetized with adjusting the inhalational flow of isoflurane while maintaining their heart rate at 450-550 beats/min and their body temperature at 36-38 °C. M-mode images of the left ventricle were obtained at papillary muscle level and used to measure Left ventricular internal diameters at end-diastole (LVIDd), left ventricular internal diameters at end-systole (LVIDs), fractional shortening (FS) and ejection fraction (EF). Three consecutive cardiac cycles were performed for analysis and the average indices (LVIDd, LVIDs, FS, and EF) were calculated for each mouse.

### Quantitative real-time RT-PCR (qRT-PCR)

qRT-PCR was performed to measure mRNA levels in heart tissues and NRCMs. The primer sequences used for RT-PCR are listed in Supplemental Table S1. Please see the Supplementary Material for more details.

### Transferase-mediated (dUTP) nick-end labelling (TUNEL) staining

Myocardial apoptosis was detected by terminal deoxynucleotidyl TUNEL staining. For more details, please refer to the Supplemental Methods.

### Immunofluorescence staining

To visualize LC3A/B and Parkin colocalization, VP1 and Mito Tracker as well as Mito Tracker and Parkin colocalization in NRCMs, immunofluorescence staining was also performed. For the observation on colocalization of lysosome and mitochondria to detect mitophagy, NRCMs were incubated with Mito Tracker Green and Lyso Tracker Red. For more details, please refer to the Supplementary Material.

### Transmission electron microscopy (TEM)

Heart tissues were fixed in 2.5% glutaraldehyde in sodium cacodylate buffer. The tissues were sliced into 50 μm sections and photographed under an electron microscope. For more details, please refer to the Supplementary Material.

### Statistics

All statistical analyses were performed with GraphPad Prism 9.0 software. All values are expressed as mean ± SEM. Statistical analyses were performed using a 2-tailed unpaired Student's t-test for two-group comparisons. For comparisons among three or more groups, statistical significance was analyzed by one-way ANOVA with Tukey's multiple-comparison test. Before the ANOVA, homogeneity among the variances was tested. Statistical significance was set at *P* < 0.05.

## Results

### During VMC, Parkin and BNIP3-mediated mitophagy is acutely upregulated

**Figure 1:**
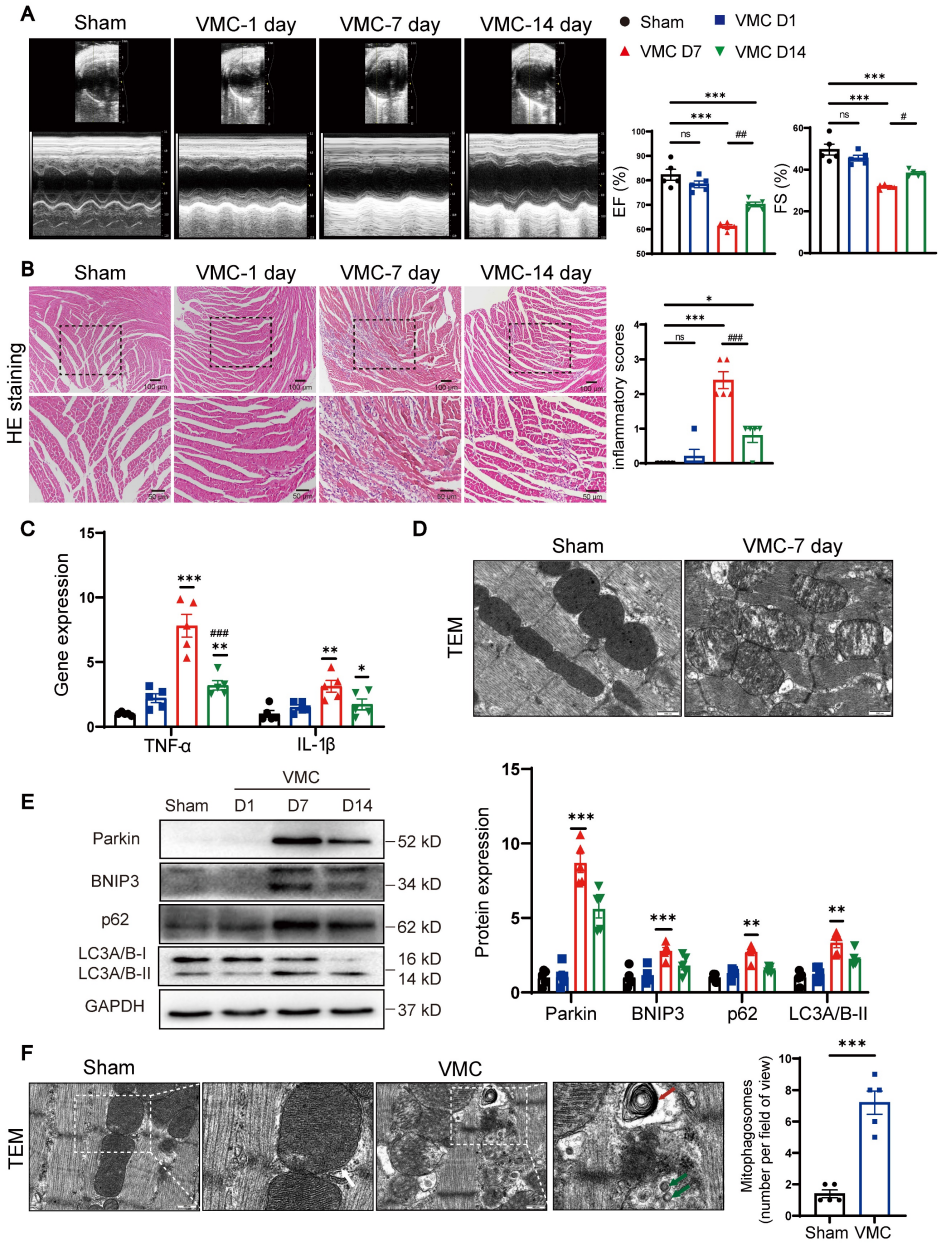
** During VMC, Parkin and BNIP3-mediated mitophagy is acutely upregulated. (A)** Representative photographs of M-mode echocardiography of the left ventricle (LV) were shown. Quantitative data of ejection fraction (EF) and fractional shortening (FS). n = 5.** (B)** Histological alterations in mouse hearts were analyzed by HE staining and the scores of inflammatory cell infiltration were quantified. n = 5.** (C)** The levels of TNF-α and IL-1β in mouse hearts were determined by qPCR. **(D)** Mitochondrial morphology changes in mouse hearts were analyzed by TEM. Scale bar = 500 nm. n = 5.** (E)** Western blot analysis of p62, Parkin, BNIP3 and LC3A/B-I/II expression in heart tissues. **(F)** TEM images to detect mitophagy in heart tissues and the quantitative analysis of mitophagosomes in the indicated groups. The zoomed-in images represent high-magnification views of the outlined areas. Green arrows indicate mitophagosomes, red arrows indicate mitophagic/autophagic multi-lamellar vesicles and white arrows indicate normal mitochondria. Scale bar = 400 nm. n = 5. Data represent the mean ± SEM. **P < 0.01, and ***P < 0.001 vs. Sham group.* #P* < 0.05,* ##P* < 0.01, *##P* < 0.001 vs. VMC D7 group.

We first observed that ejection fraction and fractional shortening of cardiac functions were significantly decreased at 7 days post-VMC, compared with Sham group (Figure 1A). Consistent with our previous findings 11, HE staining revealed that the inflammatory scores peaked at 7 days post-VMC and declined partially by 14 days (Figure 1B). Subsequently, we examined the levels of inflammatory factors in whole mouse heart tissues. TNF-α and IL-1β expression levels were markedly increased at 7 days (Figure 1C). Moreover, we examined mitochondrial ultrastructural changes by TEM. In Sham mice, the mitochondria had prominent cristae and an intact membrane, whereas abnormal mitochondrial swelling with blurred cristae were observed in VMC mice (Figure 1D). To further explore whether mitophagy was pathologically relevant to the progression of CVB3-induced myocarditis, we assessed mitophagy protein levels in mouse hearts. Western blot assay revealed that Parkin levels reached maximal expression at 7 days post-VMC. BNIP3, a mitophagy receptor protein 14, was also induced and accumulated at 7 days. Next, to evaluate autophagic flux, p62 and LC3A/B-II were quantified. P62 and LC3A/B-II levels were increased at 7days, indicating the initial activation of autophagy and subsequent blockage of autophagic flux (Figure 1E). In line with this view, TEM results revealed an increased number of mitophagosomes in VMC mouse hearts (Figure 1F). Together, these data suggest Parkin- and BNIP3-mediated mitophagy are associated with the regulation of cardiac inflammation in VMC, which leads to mitophagosome accumulation. Based on the degree of cardiac inflammation, VMC-7day animal model was used in all subsequent studies.

### CVB3 induced Parkin-mediated mitophagy and blocked autophagy flux in NRCMs

**Figure 2:**
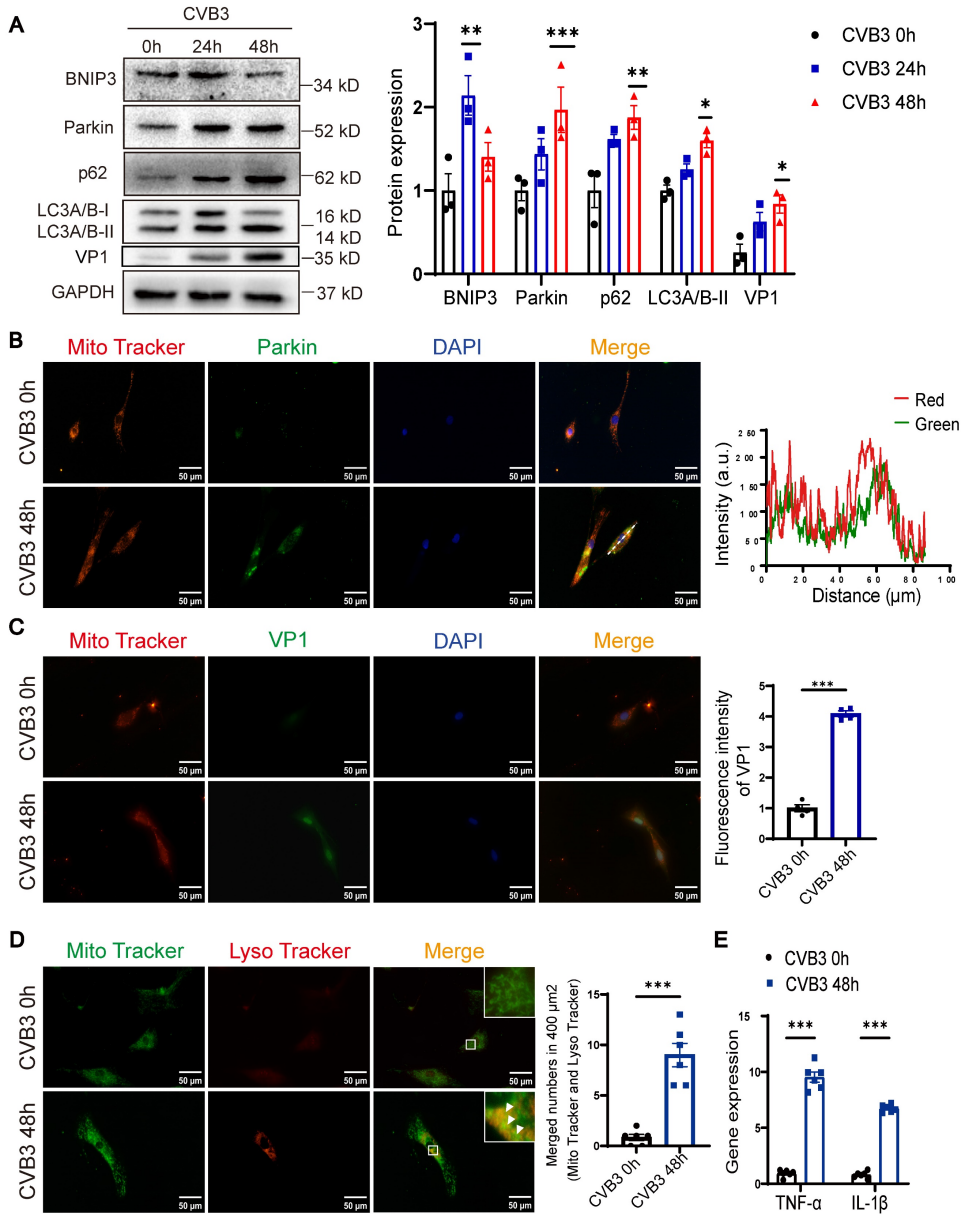
** CVB3 induced Parkin-mediated mitophagy and blocked autophagy flux in NRCMs.** NRCMs were infected with CVB3 at a multiplicity of infection (MOI) of 10. **(A)** Western blot analysis of p62, Parkin, BNIP3, LC3A/B-I/II and VP1 expression in NRCMs infected with CVB3. n = 3.** (B)** Immunofluorescence staining to examine the colocalization Parkin (green) and Mito Tracker (red). Scale bar = 50 μm. Intensity profiles were obtained using ImageJ software, along the dashed line. **(C)** Immunofluorescence staining to examine the colocalization VP1 (green) and Mito Tracker (red). Scale bar = 50 μm. Quantification of mean optical density values of VP1. **(D)** Microscopy of cells double stained with Lyso Tracker for lysosomes (red) and Mito Tracker for mitochondria (green). Scale bar = 50 μm. Count of cells with Lyso Tracker on mitochondria (merged signal, yellow) in 400 μm^2^. n = 6.** (E)** The levels of TNF-α and IL-1β in NRCMs were determined by qPCR. n = 6. Data represent the mean ± SEM. *P < 0.05, **P < 0.01 vs. CVB3 0 h group.

To examine the effects of CVB3 on cardiomyocytes, CVB3 was used to stimulate NRCMs. The steady-state level of LC3-II was elevated at 24 h post-CVB3 infection, and this lasted for 48 h (Figure 2A). Interestingly, the abundance of Parkin showed similar dynamic changes. Activation of autophagic flux or downstream blockade of autophagy vacuoles may lead to increasing the levels of LC3-II. The turnover of p62 is widely used to monitor autophagic flux. P62 levels were also elevated at 24 h and remained above baseline on the following day, which likely reflects a decrease in autophagic flux. BNIP3 expression was upregulated at 24 h but downregulated subsequently. Moreover, the VP1 expression was increased at 48 h (Figure 2A). In the NRCMs, a merge of signals from Parkin and Mito Tracker was observed at 48 h (Figure 2B). Similarly, the co-localization of LC3 and Parkin was also apparent at 48 h post-infection compared to control group (Figure S1A). VP1 was localized in both cytoplasm and the nucleus, but with the infected time growing, it transferred into the nucleus 15. The IF assay showed enhanced co-staining for VP1, Mito Tracker and DAPI, indicating CVB3 infection caused nuclear retention of VP1 in NRCMs (Figure 2C). Mito Tracker Green and Lyso Tracker Red staining showed that CVB3 infection increased the overlay of mitochondria and lysosomes (Figure 2D).

To better assess lysosomal activity, we utilized mock-infected 48 h NRCMs as a control. The results showed that the fluorescence intensity of Lyso Tracker staining was reduced in CVB3 group, suggesting that CVB3 infection led to an increase in lysosome acidification but not sufficient to maintain it (Figure S1B). Chloroquine (CQ) treatment significantly decreased this colocalization of Mito Tracker and Parkin and increased the level of LC3-II, suggesting that autophagic turnover was blocked. (Figure S1C-D). Besides, qRT-PCR results showed that CVB3 infection increased TNF-α and IL-1β expression in NRCMs (Figure 2E). In summary, the data suggest that Parkin-mediated mitophagy is involved in the pathogenesis of VMC, resulting in a blockade at the late stages of autophagosome maturation.

### Knockdown of Parkin alleviated mitophagy and inflammation of cardiomyocytes

**Figure 3:**
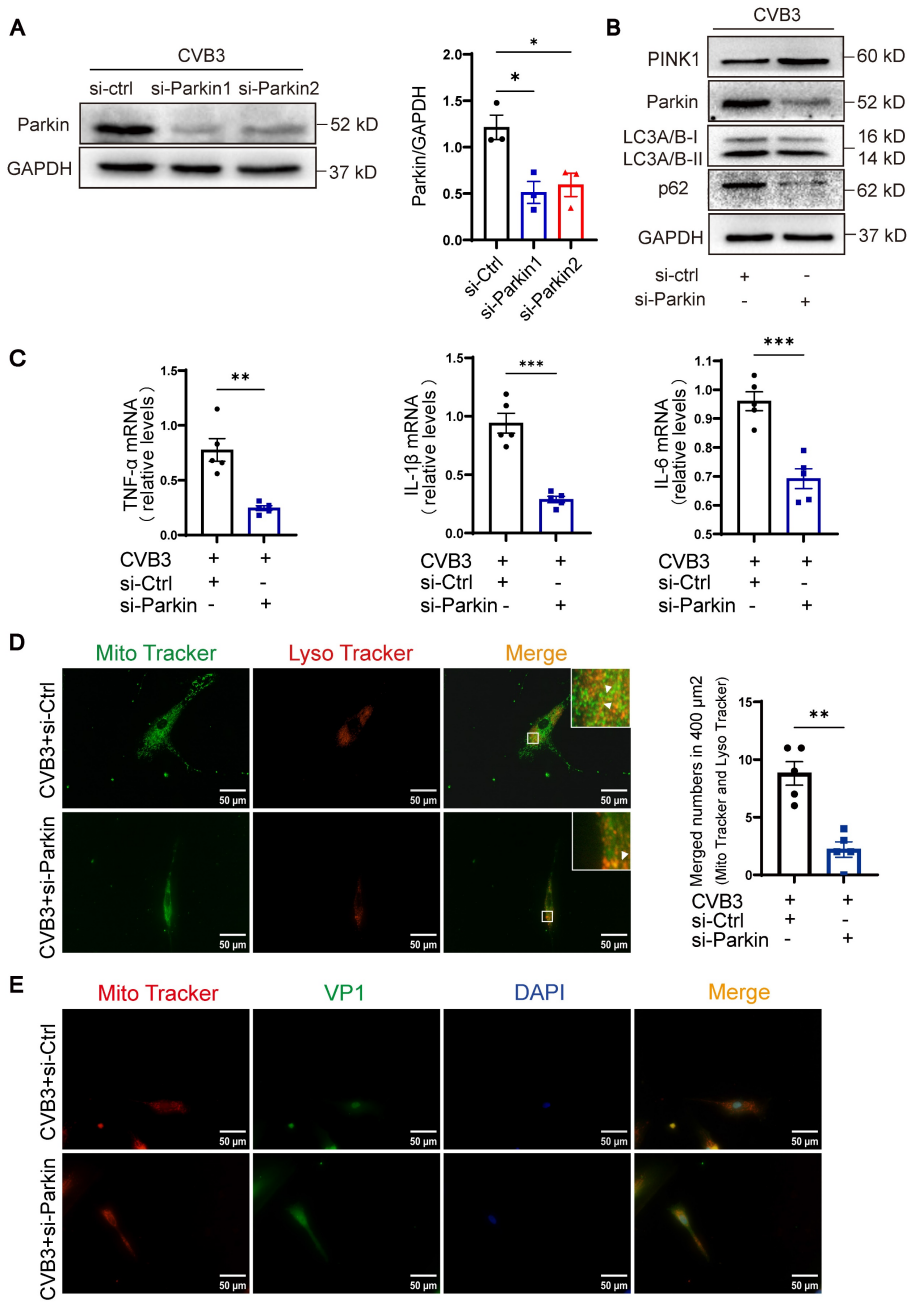
** Knockdown of Parkin alleviated mitophagy and inflammation of cardiomyocytes. (A)** Western blot analysis of Parkin silencing effects in NRCMs infected with CVB3. n = 3. **(B)** Western blot analysis of PINK1, Parkin, p62 and LC3A/B-I/II expression in NRCMs treated in CVB3 with si-Parkin or si-Ctrl. **(C)** The levels of TNF-α, IL-1β and IL-6 in NRCMs were determined by qPCR. n = 5. Data represent the mean ± SEM. *P < 0.05, **P < 0.01, and ***P < 0.001 vs. CVB3+si-Ctrl group. **(D)** Microscopy of cells double stained with Lyso Tracker for lysosomes (red) and Mito Tracker for mitochondria (green). Scale bar = 50 μm. Count of cells with Lyso Tracker on mitochondria (merged signal, yellow) in 400 μm^2^. n = 5. **(E)** Immunofluorescence staining to examine the colocalization VP1 (green) and Mito Tracker (red). Scale bar = 50 μm. n = 3.

To further investigate the function of Parkin-mediated mitophagy during the pathological activation of cardiomyocytes in vitro, we used siRNAs (small interfering RNAs) targeting back-splicing of Parkin to specifically knockdown Parkin expression. The silencing effect of si-Parkin1 was about 50% in the NRCMs infected with CVB3, compared to si-Ctrl (Figure 3A). Si-Parkin1 was used for subsequent experiments. Knockdown of Parkin in NRCMs by siRNA resulted in a mild reduction in LC3A/B-II and p62, with a significant increase in the protein level of PINK1, indicating that mitophagy activity was decreased (Figure 3B). We found knockdown of Parkin significantly decreased the levels of inflammatory cytokines in NRCMs, including TNF-α, IL-1β and IL-6 (Figure 3C). Moreover, knockdown of Parkin also elicited a decrease of mitophagy in NRCMs and induced the transfer of a larger proportion of cytoplasm-localized VP1 to the nuclear, concurrent with higher nuclear levels of VP1. (Figure 3D-E). These findings imply that impaired mitophagy is associated with the activation of cardiomyocyte inflammation caused by CVB3 in vitro. However, Parkin knockdown effectively attenuated these effects and hence facilitated viral replication.

### Parkin silencing worsened mortality and cardiac dysfunction during VMC

**Figure 4:**
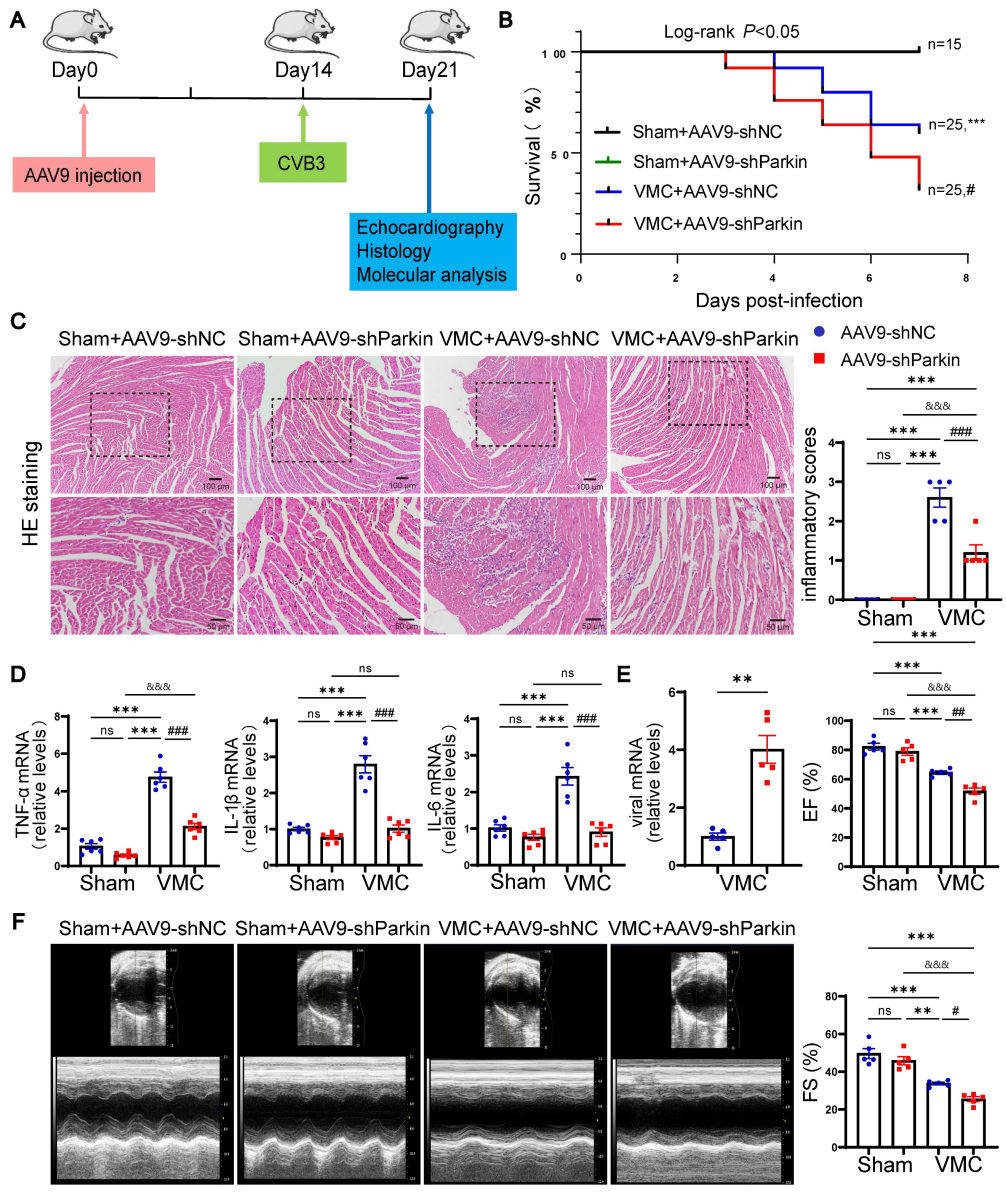
** Parkin silencing worsened mortality and cardiac dysfunction during VMC. (A)** Sham mice and VMC mice were treated with AAV9-shNC and AAV9-shParkin by intravenous injection into the tail two weeks ago, and after an additional one week, mice were euthanized. **(B)** The survival rate was monitored daily until day 7. n = 15-25. **(C)** Histological alterations in mouse hearts were analyzed by HE staining and the scores of inflammatory cell infiltration were quantified. **(D)** The levels of TNF-α, IL-1β and IL-6 in mouse hearts were determined by qPCR. **(E)** The levels of viral mRNA in mouse hearts were determined by qPCR. **(F)** Representative photographs of M-mode echocardiography of the left ventricle (LV) were shown. Quantitative data of ejection fraction (EF) and fractional shortening (FS). Data represent the mean ± SEM. n = 5. ns *P* > 0.05, *P < 0.05, **P < 0.01, and ***P < 0.001 vs. Sham+AAV9-shNC group. #*P* < 0.05, ##*P* < 0.01, ###*P* < 0.001 vs. VMC+AAV9-shNC group. &&&*P* < 0.001 Sham+AAV9-shParkin group vs. VMC+AAV9-shParkin group.

**Figure 5:**
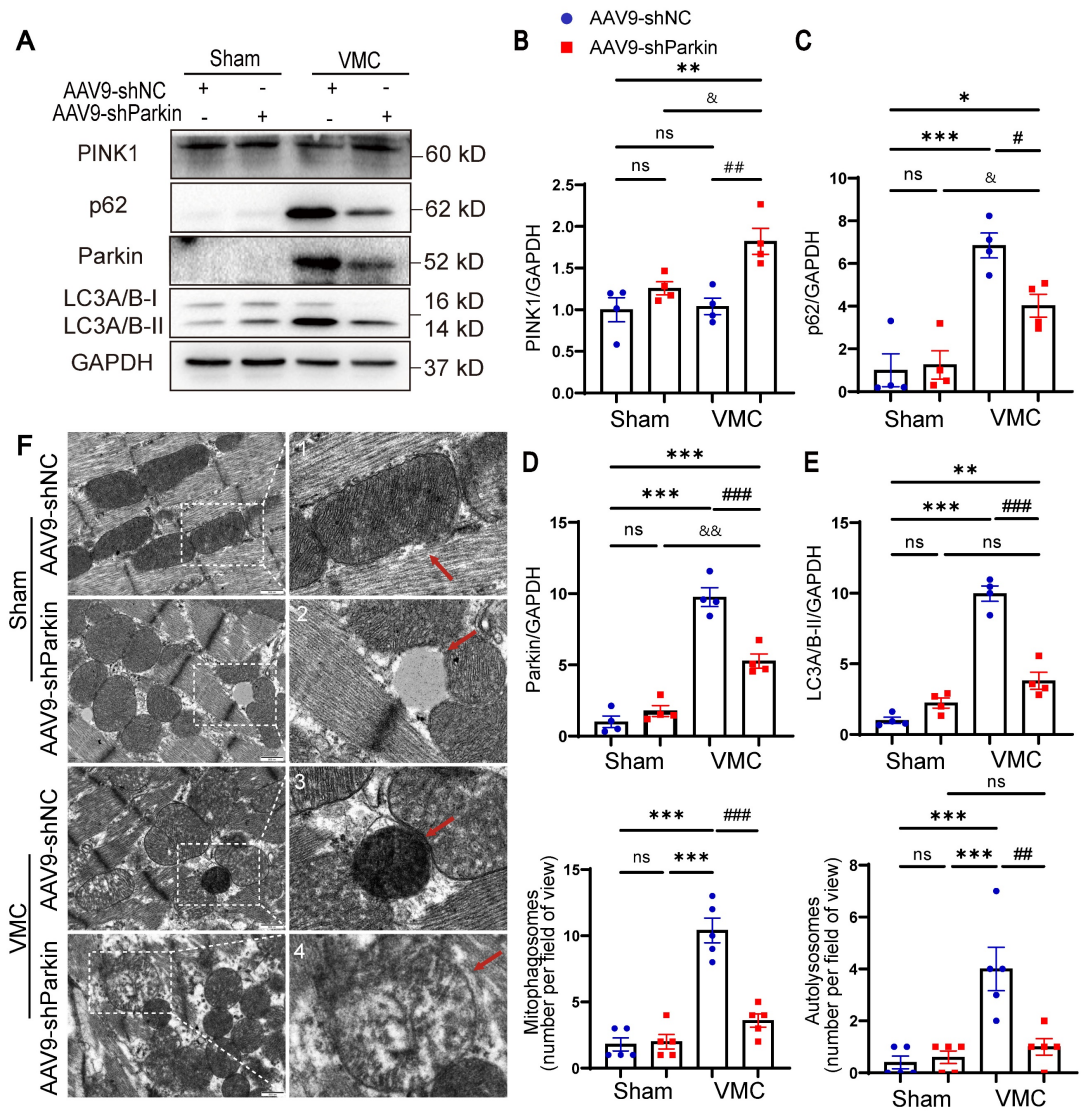
** Parkin silencing reduced the mitophagy activity during VMC. (A-E)** Western blot analysis of PINK1, Parkin, p62 and LC3A/B-I/II expression in heart tissues from Sham mice and VMC mice with Parkin silencing. n = 4. Data represent the mean ± SEM. ns *P* > 0.05, *P < 0.05, **P < 0.01, and ***P < 0.001 vs. Sham+AAV9-shNC group. #*P* < 0.05, ##*P* < 0.01, ###*P* < 0.001 vs. VMC+AAV9-shNC group. &*P* < 0.05 and &&*P* < 0.01 Sham+AAV9-shParkin group vs. VMC+AAV9-shParkin group. **(F)** TEM images showing the changes of mitophagosomes and autolysosomes in the hearts. Approximately 15-20 random fields with 2000-2500 mitochondria were analyzed per heart sample. Scale bar = 500 nm. n = 5. The zoomed-in images represent high-magnification views of the outlined areas. Red arrows indicate (1) normal mitochondria, (2) electron lucent vacuoles, (3) autolysosomes, (4) damaged mitochondria.

To study the potential role of Parkin-mediated mitophagy during the period of acute VMC, we constructed shParkin and negative control (shNC) AAV9 particles. Then, we employed tail vein injection of AAV9 to specific silence of Parkin in mouse hearts (Figure 4A). By measuring EGFP signals, the transduction efficiency of AAV9-shParkin was found to be approximately 70%. Additionally, qRT-PCR assays and western blotting verified that the injection of AAV9-shParkin dramatically decreased Parkin expression in cardiomyocytes compared to controls (Figure 5B and Figure S2A-B). We sought to determine whether Parkin silencing could prevent cardiac dysfunction and inflammation in response to CVB3-induced myocarditis. Compared to the Sham+AAV9-shNC group, there was no significant difference in the survival rates of Sham+AAV9-shParkin mice. Surprisingly, the 7-days mortality in the VMC+AAV9-shNC group was 40%, while it was 68% in VMC+AAV9-shParkin mice (log-rank *P* < 0.05, Figure 4B). The inflammatory response was markedly lower in the hearts of AAV9-shParkin mice than in the AAV9-shNC mice at 7 days post-VMC (Figure 4C). At the same time, the mRNA levels of the inflammatory cytokines TNF-α, IL-6 and IL-1β were significantly lower in the VMC+AAV9-shParkin group (Figure 4D). Moreover, we found Parkin silencing could produce more cardiac viral loads, compared with the control group (Figure 4E). Echocardiography showed that resting EF and FS were similar in Sham+AAV9-shNC and Sham+AAV9-shParkin mouse hearts. However, EF and FS values were lower in the VMC+AAV9-shParkin group than in the VMC+AAV9-shNC group after VMC (Figure 4F). These data provide direct evidence that Parkin-dependent mitophagy is required for the cardiac inflammatory effects of VMC, suggesting that changes in mouse myocardial Parkin expression levels under normal conditions exert no effect on the survival rate and heart morphology and function. Parkin silencing aggravates VMC-induced contractile dysfunction and mortality, although it reduces inflammatory responses.

### Parkin silencing reduced the mitophagy activity during VMC

To confirm the effect of Parkin-mediated mitophagy in vivo, we examined the expression of mitophagy markers. VMC+AAV9-shParkin mice elicited a distinct decrease in the levels of LC3A/B-II and p62 compared to VMC+AAV9-shNC mice. However, PINK1 protein levels were mildly increased in the hearts of CVB3-exposed AAV9-shParkin mice, suggesting that, to some extent, PINK1 levels may also reflect the accumulation of damaged mitochondria (Figure 5A-E). Next, whether Parkin silencing altered mitophagy was investigated. Analysis of TEM changes of mitophagy revealed that VMC+AAV9-shNC mice showed an increase in mitophagosomes and autolysosomes in the hearts, whereas the number of mitophagosomes and autolysosomes was reduced in VMC+AAV9-shParkin mice (Figure 5F), indicating that the overall mitophagy activity in the hearts decreased. Insufficient mitochondrial clearance leads to accumulation of damaged mitochondria. Moreover, VMC+AAV9-shParkin mice exhibited more severe myocardial structural disorders as well as mitochondrial damage, including mitochondrial swelling and increased number of mitochondrial vacuolation (Figure 5F). Taken together, these data suggest that the Parkin silencing results in a low level of mitophagy activity in the hearts which contributes to the negative pathological remodeling of the myocardium with cardiac dysfunction.

### Parkin silencing exacerbated apoptosis without activating the NF-κB-related pathway

**Figure 6:**
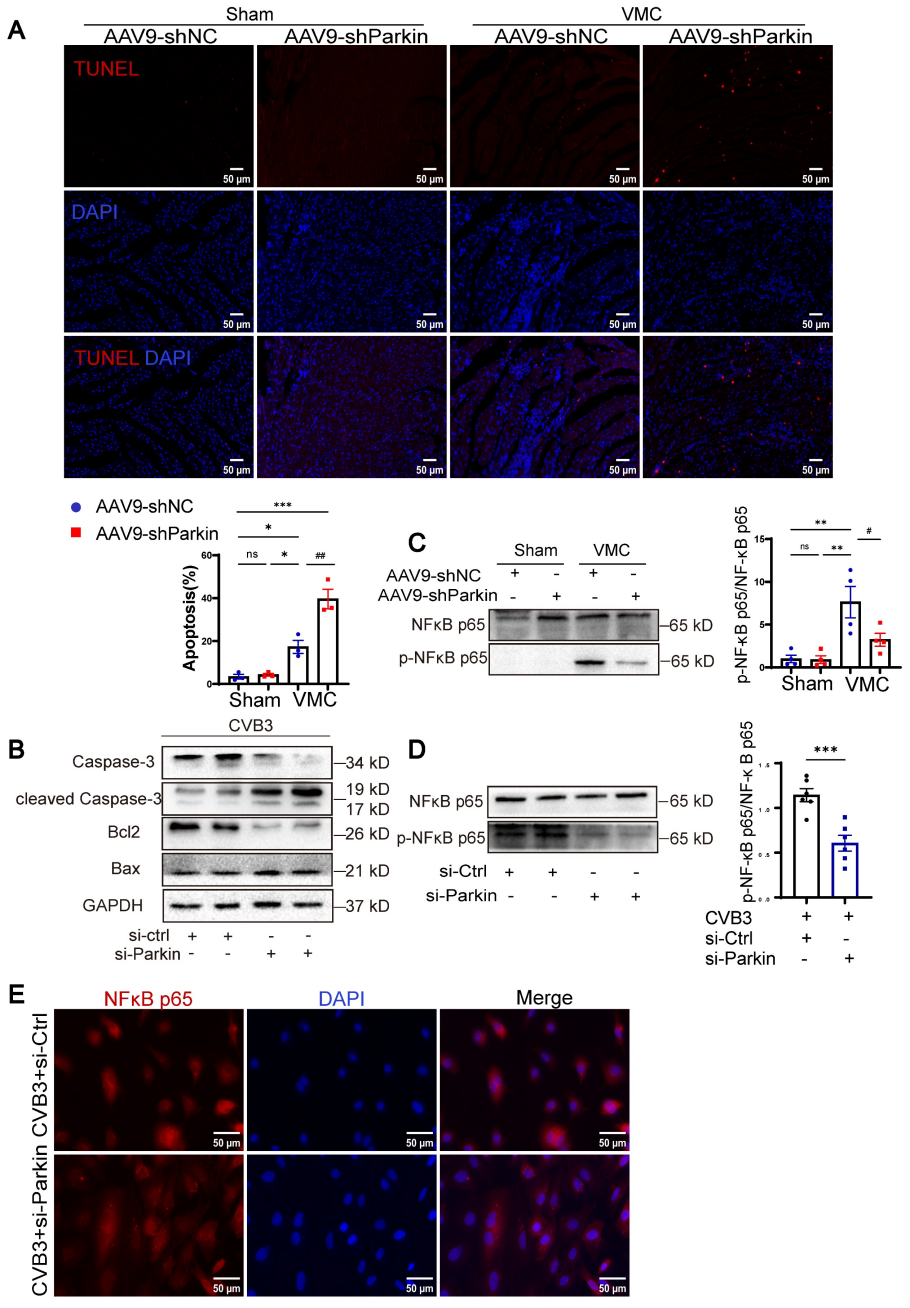
**Parkin silencing exacerbated apoptosis without activating the NF-κB-related pathway. (A)** Cardiomyocyte apoptosis in heart tissues were measured by TUNEL staining. Scale bar = 50 μm. n = 3.** (B)** Western blot analysis of Caspase-3, cleaved-Caspase-3, Bcl2 and Bax expression in NRCMs treated in CVB3 with si-Ctrl or si-Parkin. n = 3.** (C)** Western blot analysis of NF-κB p65 and phospho-NF-kB p65(Ser536) expression in each group of mouse hearts. Data are presented as mean ± SEM. n = 6. ns *P* > 0.05 and **P < 0.01 vs. Sham+AAV9-shNC group. #*P* < 0.05 vs. VMC+ AAV9-shNC group. **(D)** Western blot analysis of NF-κB p65 and phospho-NF-kB p65(Ser536) expression in NRCMs. ***P < 0.001 vs. CVB3+si-Ctrl group. **(E)** Immunofluorescence staining to examine the colocalization NF-κB p65 (red) and nucleus (DAPI, blue). Scale bar = 50 μm. n = 3.

Since CVB3 can induce mitochondrial dysfunction resulting in cardiomyocyte apoptosis, we sought to investigate the effect of Parkin-mediated mitophagy on it 16. The results of TUNEL staining are shown in Figure 6A. The number of TUNEL-positive cardiomyocyte nuclei in VMC+AAV9-shNC mice was greater than that in Sham+AAV9-shNC and Sham+AAV9-shParkin mice, whereas VMC+AAV9-shParkin increased apoptosis, which is consistent with the accumulation of damaged mitochondria. The levels of cleaved-Caspase-3 and Bax increased in NRCMs treated with CVB3+si-Parkin, whereas the levels of Caspase-3 and Bcl2 decreased compared with those in the CVB3+si-Ctrl group (Figure 6B). NF-kB signaling is a major driver of inflammation 17. In VMC, the inflammatory response via the NF-κB pathway is a key contributor to secondary myocardial injury 18, 19. To further examine the underlying pathways and mechanisms that are responsible for the cardiac inflammation and mitophagy of Parkin silencing mice after VMC, we additionally assess the protein level of NF-κB p65 and phospho-NF-κB p65. VMC-induced upregulation of NF-κB p65 and the downstream effectors of NF-κB p65 phosphorylation, were significantly reversed by Parkin silencing (Figure 6C). Similar results were observed in vitro (Figure 6D). The immunofluorescence assay also showed that CVB3 infection with si-Ctrl transfection induced nuclear translocation of NF-κB p65, which was predominantly localized in the cytosol under normal conditions. Furthermore, NRCMs transfected with CVB3+si-Parkin, attenuated nuclear translocation of NF-κB p65 (Figure 6E). These findings demonstrated that the anti-inflammatory impacts of Parkin silencing in cardiomyocytes were primarily connected to its suppression of the NF-κB pathway. In summary, Parkin silencing exerted anti-inflammatory effects that mainly related to its inhibition on NF-κB pathway and significantly increased VMC-induced apoptosis with the accumulation of damaged mitochondria.

## Discussion

Despite the use of pharmacological agents to modify VMC, clinical treatments specifically for myocarditis progression are not available. In the present study, we first provide direct evidence that augmenting VMC induces Parkin-mediated mitophagy during the acute phase, which is partially mediated by a BNIP3-dependent mechanism. And we further determine to the role of Parkin-mediated mitophagy in the heart during VMC. We demonstrated that (1) Under conditions of severe CVB3 infection, there were clear pathogenic signs of VMC on day 7, manifested as a decline in cardiac function, elevation in inflammation, and evidence of mitochondrial damage. (2) CVB3 infection in vivo and in vitro resulted in Parkin-mediated mitophagy and blocked autophagic flux by inhibiting the fusion of autophagosomes with lysosomes. (3) Parkin silencing in cardiomyocytes not only reduced cardiac function and survival rate, but also inhibited the overall mitophagy activity with the accumulation of damaged mitochondria. However, it inhibited the inflammatory response via suppression of the NF-κB pathway, suggesting that it may increase the persistence of virus infection and enhance the development of VMC.

Growing evidence has shown that mitochondria play diverse roles in innate immune response, including the functions as signaling hubs, facilitating crosstalk between energy and metabolites of inflammation, and regulating the immune response 20. Mitophagy aims to regulate mitochondrial quality control by removing mitochondria via the autophagy/lysosomal pathway 21. Mitophagy is a dynamic process essential for the maintenance of cardiac function. Changes in mitophagy activity can exacerbate or mitigate cardiac pathophysiology, including mitochondrial depolarization, reduced oxygen consumption and decreases in cardiomyocyte contraction 22. However, current studies are still limited to cell models of CVB3 infection without sufficient data to assess mitophagy under physiological or pathophysiological conditions during VMC. This study insights into the role of Parkin-mediated mitophagy in vivo response to VMC. CVB3 infection impaired autophagic flux as previously reported 23. Our investigation showed that during VMC, PINK1-Parkin-mediated mitophagy persists to protecting cardiomyocytes under mitochondrial stress conditions. However, continuous mitophagy inevitably imposes an additional burden on lysosomes (as manifested by the accumulation of LC3A/B-II and p62), leading to impaired autophagic flux. It has been previously reported that CVB3 disrupts host lysosomal function and promotes viral replication by targeting TFEB for protein hydrolysis 24. CVB3 restricts protein degradation in the mitophagic process, possibly to prevent the destruction of viral particles, drive lysosome-dependent viral release and utilize mitophagy-associated lipids to generate new virus particle 25, 26.

Parkin is an E3 ubiquitin ligase that regulates mitophagy in hearts 27. Although not essential for basic mitochondrial homeostasis, Parkin appears to be necessary for adaptation to stress and altered energy metabolism. For example, the absence of Parkin-mediated mitophagy in perinatal mice renders them unable to tolerate the transition of fatty acid oxidation after birth, which contributes to premature death. Parkin silencing accentuates HFD-induced cardiac anomalies 22, 28, 29. Although these roles of Parkin have previously reported, to the best of our knowledge, the present study is the first to demonstrate the effects of Parkin-mediated mitophagy in vivo on VMC-associated cardiac damage. The present study suggests that Parkin silencing alleviates cardiac inflammation via inhibition of the NF-κB pathway, as shown by attenuated cytokines in the heart. Surprisingly, it has certain limitations, since in response to serious CVB3 infection, overwhelming cardiac dysfunction and injury still occurred in AAV9-shParkin mice, even with low levels of mitophagy activity. We also found that the levels of PINK1 were upregulated, and apoptosis increased in areas of myocardial injury with rare inflammatory cell infiltration in VMC+AAV9-shParkin mice. Even if PINK1 accumulation may indicate that an increase in mitophagy activity, increased stabilized PINK1 protein levels can also be considered as the accumulation of damaged mitochondria regardless of mitophagy status 30. Thus, decreased mitophagy activity remains, at least in part, a leading cause of heart function deterioration in AAV9-shParkin mice.

NF-κB has been reported to be a key signaling pathway in the inflammatory response and contributes to the transcription of inflammatory cytokines 31. The accumulation of p62 has been reported to activate NF-κB through its TRAF6 binding motif 32. However, recent studies have shown p62 may also inhibit TRAF6 signaling 33. Despite evidence implicating the cleavage of p62 results in disrupted selective autophagy and impaired NF-κB signaling during the early stage of CVB3 infection 34. Subsequently, Yasir Mohamud et al demonstrated that p62 plays an antiviral role in a low dose of CVB3 infection, which addressed the limitations of previous research 35. And TRAF6 silencing resulted in a decrease of CVB3 replication, which may vary due to different settings of CVB3 infection 36. Furthermore, Olivia et al showed that the recruitment of NF-κB effector NEMO may activate Parkin-mediated mitophagy and initiate inflammatory signaling, causing the clearance of autophagosomes more slowly 37. We found that p62 levels increased and the autophagic flux was impaired during acute VMC, which activated the NF-κB pathway. Thus, we suspected that Parkin silencing was defective in mitochondrial p62 recruitment, which further inhibited the NF-κB pathway. Taken together, the role of p62, certainly further studies are warranted to determine the effects of different viral loads and time-dependent strategy on NF-κB levels or activity. Notably, NF-κB pathway plays important roles in cell survival that promotes the expression of anti-apoptotic proteins, like Bcl-2 family members 38. It is uncertain whether the decrease in mitophagy itself contributes to the development of cardiac dysfunction in VMC, which may trigger the overall deterioration of mitochondria and contribute to severe cardiac dysfunction through multiple pathways. And signals between mitophagy and inflammation occur dynamically during the development of diverse diseases, highlighting the need for further studies.

As a cell death factor, BNIP3 belongs to the Bcl2 pro-apoptotic family. Recently, BNIP3 was reported to regulate mitophagy. However, the mechanism through with VMC modulates the BNIP3 pathway remains unclear. According to other studies, BNIP3 plays a critical and dual role in mitochondria 39. On the one hand, BNIP3 facilities mitochondrial depolarization and delivers mitochondria to autophagosomes to initiate mitophagy during hypoxia. On the other hand, it also leads to mitochondrial dysfunction, followed by the induction of cell death through the activation of the proapoptotic protein Bax/Bak 40. Therefore, its effect on mitochondria might be an outcome of the balance between its dual roles. Our data suggest that mitophagy is partially activated by BNIP3 signaling during VMC.

This study had some limitations. First, p62 was not as reliable as LC3 in reflecting autophagic flux in the hearts. Consequently, depending solely on the abundance of this protein may not be sufficient to confirm autophagic flux. It is essential to use several approaches to examine autophagic flux. Second, we did not detect these influences of Parkin in the end-stage of VMC. Thus, the protection of Parkin cannot be directly extended to these conditions. Besides, cardiomyocyte-specific knockout mice of Parkin should be added to further confirm the roles of Parkin inhibition in VMC. Third, the regulatory mechanisms between Parkin and NF-κB need to be further studied. Additionally, the results emphasize the physiological importance of mitophagy activity for cardiomyocyte function, indicating that the application of Parkin-silenced mice in disease models should be performed with caution.

Collectively, these results clearly indicate that Parkin-mediated mitophagy was significantly increased in mouse hearts with CVB3-induced acute myocarditis, but also blocked autophagic flux at the late stages of autophagosome maturation, and that Parkin silencing to decrease the overall mitophagy activity in hearts is associated with CVB3-induced vulnerability to cardiac dysfunction and survival. In the future, therapeutic efforts to restore lysosomal function and re-activate the autophagic flux at the late stages of autophagosome maturation in cardiomyocytes will provide new insights into improving the clinical outcome of VMC.

## Supplementary Material

Supplementary methods and figures.

## Abbreviations

VMC: viral myocarditis
CVB3: Coxsackievirus B3
AAV9: adeno-associated virus 9
NRCMs: neonatal rat cardiac myocytes
PINK1: PTEN-induced putative kinase 1
shParkin: short hairpin RNA for Parkin
EGFP: enhanced green fluorescent protein
LVIDd: left ventricular internal diameters at end-diastole
LVIDs: left ventricular internal diameters at end-systole
FS: fractional shortening
EF: ejection fraction
CQ: Chloroquine
TEM: transmission electron microscopy
HFD: high fat diet

## References

[1] Magnani JW, Dec GW (2006). Myocarditis: current trends in diagnosis and treatment. Circulation.

[2] Cooper LT Jr (2009). Myocarditis. N Engl J Med.

[3] Ghigo A, Franco I, Morello F, Hirsch E (2014). Myocyte signalling in leucocyte recruitment to the heart. Cardiovasc Res.

[4] Yajima T, Knowlton KU (2009). Viral myocarditis: from the perspective of the virus. Circulation.

[5] Mohamud Y, Li B, Bahreyni A, Luo H (2023). Mitochondria Dysfunction at the Heart of Viral Myocarditis: Mechanistic Insights and Therapeutic Implications. Viruses.

[6] Sciarretta S, Maejima Y, Zablocki D, Sadoshima J (2018). The Role of Autophagy in the Heart. Annu Rev Physiol.

[7] Tong M, Sadoshima J (2016). Mitochondrial autophagy in cardiomyopathy. Curr Opin Genet Dev.

[8] Sin J, McIntyre L, Stotland A, Feuer R, Gottlieb RA (2017). Coxsackievirus B Escapes the Infected Cell in Ejected Mitophagosomes. J Virol.

[9] Oh SJ, Lim BK, Yun J, Shin OS (2021). CVB3-Mediated Mitophagy Plays an Important Role in Viral Replication via Abrogation of Interferon Pathways. Front Cell Infect Microbiol.

[10] Gu X, Li Y, Chen K, Wang X, Wang Z, Lian H (2020). Exosomes derived from umbilical cord mesenchymal stem cells alleviate viral myocarditis through activating AMPK/mTOR-mediated autophagy flux pathway. J Cell Mol Med.

[11] Yue-Chun L, Gu XH, Li-Sha G, Zhou DP, Xing C, Guo XL (2021). Vagus nerve plays a pivotal role in CD4+ T cell differentiation during CVB3-induced murine acute myocarditis. Virulence.

[12] Kumar S, Wang G, Zheng N, Cheng W, Ouyang K, Lin H (2019). HIMF (Hypoxia-Induced Mitogenic Factor)-IL (Interleukin)-6 Signaling Mediates Cardiomyocyte-Fibroblast Crosstalk to Promote Cardiac Hypertrophy and Fibrosis. Hypertension.

[13] Liu X, Li M, Chen Z, Yu Y, Shi H, Yu Y (2022). Mitochondrial calpain-1 activates NLRP3 inflammasome by cleaving ATP5A1 and inducing mitochondrial ROS in CVB3-induced myocarditis. Basic Res Cardiol.

[14] Li Y, Zheng W, Lu Y, Zheng Y, Pan L, Wu X (2021). BNIP3L/NIX-mediated mitophagy: molecular mechanisms and implications for human disease. Cell Death Dis.

[15] Wang Y, Zhao S, Chen Y, Wang T, Dong C, Wo X (2019). The Capsid Protein VP1 of Coxsackievirus B Induces Cell Cycle Arrest by Up-Regulating Heat Shock Protein 70. Front Microbiol.

[16] Bock FJ, Tait SWG (2020). Mitochondria as multifaceted regulators of cell death. Nat Rev Mol Cell Biol.

[17] Sun SC (2017). The non-canonical NF-κB pathway in immunity and inflammation. Nat Rev Immunol.

[18] Van der Heiden K, Cuhlmann S, Luong le A, Zakkar M, Evans PC (2010). Role of nuclear factor kappaB in cardiovascular health and disease. Clin Sci (Lond).

[19] Liu T, Zhang M, Niu H, Liu J, Ruilian M, Wang Y (2019). Astragalus polysaccharide from Astragalus Melittin ameliorates inflammation via suppressing the activation of TLR-4/NF-κB p65 signal pathway and protects mice from CVB3-induced virus myocarditis. Int J Biol Macromol.

[20] Marchi S, Guilbaud E, Tait SWG, Yamazaki T, Galluzzi L (2023). Mitochondrial control of inflammation. Nat Rev Immunol.

[21] Ajoolabady A, Chiong M, Lavandero S, Klionsky DJ, Ren J (2022). Mitophagy in cardiovascular diseases: molecular mechanisms, pathogenesis, and treatment. Trends Mol Med.

[22] Tong M, Saito T, Zhai P, Oka SI, Mizushima W, Nakamura M (2019). Mitophagy Is Essential for Maintaining Cardiac Function During High Fat Diet-Induced Diabetic Cardiomyopathy. Circ Res.

[23] Zhang L, Qin Y, Chen M (2018). Viral strategies for triggering and manipulating mitophagy. Autophagy.

[24] Mohamud Y, Tang H, Xue YC, Liu H, Ng CS, Bahreyni A (2021). Coxsackievirus B3 targets TFEB to disrupt lysosomal function. Autophagy.

[25] Tian L, Yang Y, Li C, Chen J, Li Z, Li X (2018). The cytotoxicity of coxsackievirus B3 is associated with a blockage of autophagic flux mediated by reduced syntaxin 17 expression. Cell Death Dis.

[26] Robinson SM, Tsueng G, Sin J, Mangale V, Rahawi S, McIntyre LL (2014). Coxsackievirus B exits the host cell in shed microvesicles displaying autophagosomal markers. PLoS Pathog.

[27] Kamienieva I, Duszyński J, Szczepanowska J (2021). Multitasking guardian of mitochondrial quality: Parkin function and Parkinson's disease. Transl Neurodegener.

[28] Wu NN, Bi Y, Ajoolabady A, You F, Sowers J, Wang Q (2022). Parkin Insufficiency Accentuates High-Fat Diet-Induced Cardiac Remodeling and Contractile Dysfunction Through VDAC1-Mediated Mitochondrial Ca(2+) Overload. JACC Basic Transl Sci.

[29] Gong G, Song M, Csordas G, Kelly DP, Matkovich SJ, Dorn GW 2nd (2015). Parkin-mediated mitophagy directs perinatal cardiac metabolic maturation in mice. Science.

[30] Hoffmann RF, Zarrintan S, Brandenburg SM, Kol A, de Bruin HG, Jafari S (2013). Prolonged cigarette smoke exposure alters mitochondrial structure and function in airway epithelial cells. Respir Res.

[31] Zhao H, Wu L, Yan G, Chen Y, Zhou M, Wu Y (2021). Inflammation and tumor progression: signaling pathways and targeted intervention. Signal Transduct Target Ther.

[32] Zhong Z, Umemura A, Sanchez-Lopez E, Liang S, Shalapour S, Wong J (2016). NF-kappaB Restricts Inflammasome Activation via Elimination of Damaged Mitochondria. Cell.

[33] Kim JY, Ozato K (2009). The sequestosome 1/p62 attenuates cytokine gene expression in activated macrophages by inhibiting IFN regulatory factor 8 and TNF receptor-associated factor 6/NF-kappaB activity. J Immunol.

[34] Shi J, Wong J, Piesik P, Fung G, Zhang J, Jagdeo J (2013). Cleavage of sequestosome 1/p62 by an enteroviral protease results in disrupted selective autophagy and impaired NFKB signaling. Autophagy.

[35] Mohamud Y, Qu J, Xue YC, Liu H, Deng H, Luo H (2019). CALCOCO2/NDP52 and SQSTM1/p62 differentially regulate coxsackievirus B3 propagation. Cell Death Differ.

[36] Mohamud Y, Xue YC, Liu H, Ng CS, Bahreyni A, Luo H (2021). Autophagy Receptor Protein Tax1-Binding Protein 1/TRAF6-Binding Protein Is a Cellular Substrate of Enteroviral Proteinase. Front Microbiol.

[37] Harding O, Holzer E, Riley JF, Martens S, Holzbaur ELF (2023). Damaged mitochondria recruit the effector NEMO to activate NF-κB signaling. Mol Cell.

[38] Mattson MP, Meffert MK (2006). Roles for NF-kappaB in nerve cell survival, plasticity, and disease. Cell Death Differ.

[39] Zhang J, Ney PA (2009). Role of BNIP3 and NIX in cell death, autophagy, and mitophagy. Cell Death Differ.

[40] Webster KA, Graham RM, Bishopric NH (2005). BNip3 and signal-specific programmed death in the heart. J Mol Cell Cardiol.

