# Supplementary material for: Parkin plays a crucial role in acute viral myocarditis by regulating mitophagy activity
Source: Theranostics. 2024 Aug 26;14(13):5303–15. doi: 10.7150/thno.97675 (PMC11388078; doi:10.7150/thno.97675)
Supplement: Supplementary file 1 — Supplementary methods and figures. [file thnov14p5303s1.pdf]

Supplement material

Cell isolation and culture

Neonatal SD rat pups (0–3 days old) were purchased from Charles River and sacrificed for cardiomyocyte extraction, as previously described [1]. Hearts were removed from the thorax after euthanizing mice by 2% isoflurane inhalation; the ventricles were finely minced, and digested in a mixed enzyme solution (0.25% trypsin and phosphate buffer saline (PBS) containing 0.1% collagenase type II) for 30-35 min. To remove cardiac fibroblasts, dispersed cells were pre-plated for 1.5 h, and the supernatant containing cardiomyocytes was separated and cells were cultured in high-glucose DMEM (Gibco) supplemented with 10% fetal bovine serum (FBS), 1% penicillin/streptomycin, 10 μM 5-Bromo-2'-deoxyuridine (5-BrdU, HY-15910, MCE) at 37 °C in a 5% CO_2 incubator. NRCMs were stimulated with 20 μM Chloroquine (CQ, HY-17589A, MCE) for 12 h before infection with CVB3 in serum-free media for 2 h at 37 °C to establish a cell model of VMC, following which they were washed with PBS and grown in culture media. Finally, the cultured cardiomyocytes were harvested for western blot and qPCR analyses.

Animal models of VMC

Virus titer was routinely determined prior to infection using a 50% tissue culture infectious dose ($TCID_{50}$) assay of Hep2 cell monolayer. To induce VMC [2], 4-week-old male BALB/c mice were intraperitoneally injected with CVB3 Nancy (10^5 $TCID_{50}$, 100 μL) as previously described, while mice in the sham group were injected with the same amount of PBS. The mice were euthanized by carbon dioxide inhalation.

Myocardial Histopathology

Heart samples were put into 4% paraformaldehyde and processed for hematoxylin-eosin (HE) staining. The percentage of inflammatory cell infiltration in each heart section was calculated blindly by two skilled technicians and severity was evaluated as follows: 0 = no lesion; 1+ ≤25% of the myocardium that is involved; 2+ = 25% to 50%; 3+ = 50 to 75%; and 4+ ≥75% [3].

Western blotting

Total protein was extracted from mouse hearts and cultured cardiomyocytes in RIPA lysis buffer, and protein concentrations were measured using a BCA protein assay kit. Western blotting was performed as described previously [4], with the following antibodies:LC3A/B (1:1000, #12741s), p62 (1:1000, #23214s), Caspase-3 (1:1000, #9662s) (these antibodies were purchased from Cell Signaling Technology); GAPDH (1:1000, 60004-1-lg) ,Caspase-3/p17/p19 (1:1000, 19677-1-AP) and Bax (1:1000, 50599-2-lg) (these antibodies were purchased from Proteintech); Parkin (1:1000, sc-32282), RELA/NF-κB p65 (1:1000, sc-8008) and BNIP3 (1:1000, sc56167) (these antibodies were purchased from Santa Cruz Biotechnology); Phospho-NF-κB p65 (Ser536) (1:1000, #AF2006 ; purchased from Affinity Biosciences); VP1 clone 5-D8/1 (1:1000, #MRO-1008LC; purchased from Dako). Autophagic flux was estimated based on the LC3B-II and p62 protein levels.

Quantitative real-time RT-PCR (qRT-PCR)

Total RNA from NRCMs or heart tissues was extracted using the TRIzol reagent (Invitrogen) and converted into cDNA. A qPCR amplification was consisted: 30 s at

95 °C, 10 s at 95 °C, and 30 s at 60 °C for 40 cycles using the CFX96 Touch real-time PCR Detection system (Bio-Rad). Then, relative gene level was calculated with $2^{-\Delta\Delta CT}$ and normalized to GAPDH as the internal reference gene. The primer sequences used for RT-PCR are listed in the Supplementary Material (Table S1).

Transferase-mediated (dUTP) nick-end labelling (TUNEL) staining

Myocardial apoptosis was detected by terminal deoxynucleotidyl TUNEL staining. Briefly, apoptotic cells were treated with red fluorescein using the In Situ Cell Death Detection Kit (Roche). The heart tissues were fixed, embedded, and sectioned as described above. Subsequently, the sections were labelled to determine the reaction, according to the manufacturer's instructions. The slides were stained with DAPI solution for 5 min to visualize apoptosis.

Immunofluorescence staining

Isolated NRCMs were cultured on cell slides, fixed with 4% paraformaldehyde for 30 min and permeabilized with 0.5% Triton X-100 in PBS for 15 min at room temperature. After blocking the non-specific binding with 5% bovine serum albumin (BSA), cell slides were incubated with the primary antibodies of LC3A/B (1:200, #12741s, Cell Signaling Technology), Parkin (1:200, sc-32282, Santa Cruz Biotechnology) and VP1 clone 5-D8/1 (1:200, #MRO-1008LC, Dako) at 4 °C overnight in a humidified chamber. The cells were rinsed in PBS three times and stained with secondary antibodies conjugated to Alexa Fluor 488 or 594 (1:200, #4408, Cell Signaling Technology) for 1 h and incubated with Mito Tracker Red CMXRos (25 nM, 40741ES50, YEASEN Biotech, China) for 30 min at 37 °C. Nuclei were visualized with

DAPI. Immunostaining was visualized under a fluorescence microscope.

For the observation on colocalization of lysosome and mitochondria, cells were incubated with Mito Tracker Green FM (20 nM, 40742ES50, YEASEN Biotech, China) and Lyso Tracker Red DND-99 (50 nM, 40739ES50, YEASEN Biotech, China) at 37 °C for 30 min, and the cells were washed twice with PBS. Using a fluorescence microscope, fluorescence intensity was detected with excitation and emission wavelengths of 490 and 516 nm, respectively.

Transmission electron microscopy (TEM)

Heart tissues were cut into 1mm^3 segments and fixed in 2.5% glutaraldehyde in sodium cacodylate buffer at 4 °C for 2 h and then dehydrated through a graded ethanol series and embedded in epoxy resin at room temperature for 4 h. Tissues were then sliced using a diamond slicer and ultramicrotome (Leica, UC7), then photographed under an electron microscope (Hitachi, Japan).

The primer sequences

Table S1

**Gene**	**Forward primer (5'-3')**	**Reverse primer (5'-3')**
GAPDH (rat)	TCCCATTCTTCCACCTTTGA	ATGTAGGCCATGAGGTCCAC
IL-1β (rat)	CAGGAAGGCAGTGTCACTCA	AAAGAAGGTGCTTGGGTCCT
TNF-α (rat)	GAGGTCAACCTGCCCAAGTA	GCTGGGTAGAGAACGGATGA
IL-6 (rat)	AAGCCAGAGTCATTCAGAGCAA	GTCTTGGTCCTTAGCCACTCCT
GAPDH (mouse)	ACGGCAAATTCAACGGCACAG	ACACCAGTAGACTCCACGACATAC
IL-1β (mouse)	CACCTCACAAGCAGAGCACAAG	GCATTAGAAACAGTCCAGCCCATAC
TNF-α (mouse)	GAAGACACCATGAGCACAGAAAGC	GCCACAAGCAGGAATGAGAAGAG
Parkin (mouse)	TCATCTGGTGCCTCCGTGTG	AGTCAATGCTGCCGTTGGAAG

IL-6 (mouse)	GCTAAGGACCAAGACCATCCAAT	GGCATAACGCACTAGGTTTGC
CVB3	CACCGGATGGCCAATCCA	GCGAAGAGTCTATTGAGCTA
siParkin-1	GGAACAACAGAGTATCGTT	
siParkin-2	CCATCACTTCAGGATCCTT	

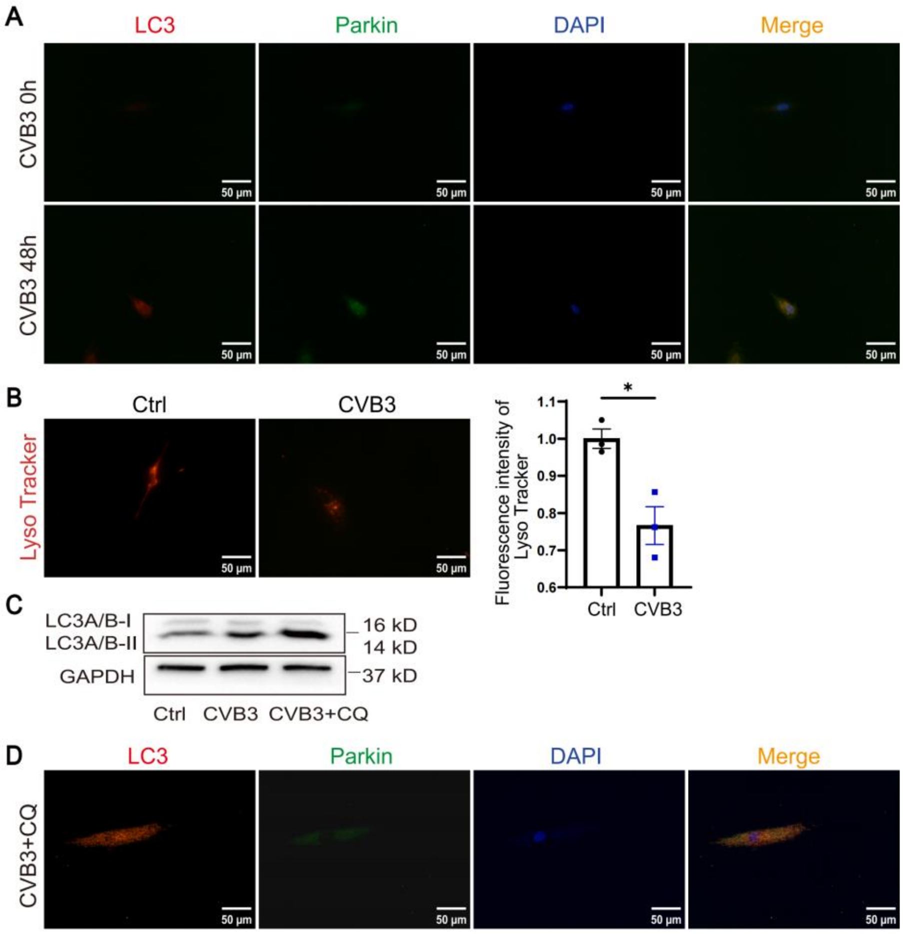

Supplementary Figure 1. CQ impaired CVB3-induced mitophagy.

(A) Immunofluorescence staining to examine the colocalization Parkin (green) and LC3 (red). Scale bar = 50 μm. **(B)** Microscopy of cells stained with Lyso Tracker for lysosomes (red) and quantification of mean optical density values. Scale bar = 50 μm. **(C)** Western blot analysis of LC3A/B-I/II expression in cultured NRCMs infected with

CVB3. **(D)** Immunofluorescence staining to examine the colocalization Parkin (green) and LC3 (red). Scale bar = 50 μm. Data represent the mean ± SEM. n = 3. *P < 0.05 vs. Ctrl group.

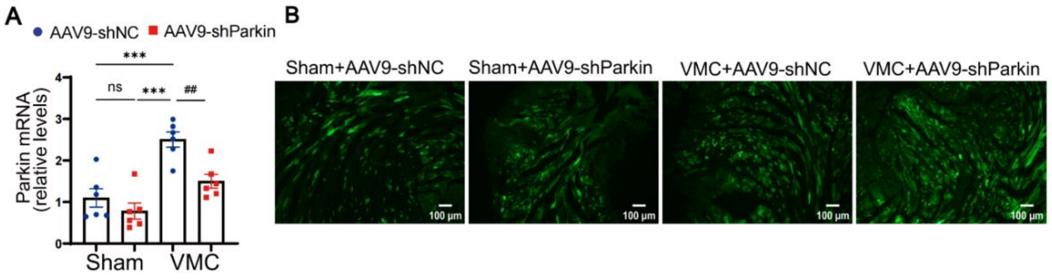

Supplementary Figure 2. Verification of AAV9 silencing (sh) Parkin in Mouse Hearts.

(A) The levels of Parkin in mouse hearts were determined by qPCR. Data represent the mean ± SEM. n = 5. *ns P* > 0.05 and **P < 0.01 vs. Sham+AAV9-shNC group. #P < 0.05 vs. VMC+ AAV9-shNC group. **(B)** The results of frozen sections showing adeno-associated virus9 (AAV9) carrying the shParkin or shNC plasmid successfully infected the heart. Scale bar =100 μm.

References

1. Kumar S, Wang G, Zheng N, Cheng W, Ouyang K, Lin H, et al. HIMF (Hypoxia-Induced Mitogenic Factor)-IL (Interleukin)-6 Signaling Mediates Cardiomyocyte-Fibroblast Crosstalk to Promote Cardiac Hypertrophy and Fibrosis. Hypertension. 2019; 73: 1058-70.
2. Gu X, Li Y, Chen K, Wang X, Wang Z, Lian H, et al. Exosomes derived from

umbilical cord mesenchymal stem cells alleviate viral myocarditis through activating AMPK/mTOR-mediated autophagy flux pathway. J Cell Mol Med. 2020; 24: 7515-30.

3. Li-Sha G, Xing-Xing C, Lian-Pin W, De-Pu Z, Xiao-Wei L, Jia-Feng L, et al. Right Cervical Vagotomy Aggravates Viral Myocarditis in Mice Via the Cholinergic Anti-inflammatory Pathway. Front Pharmacol. 2017; 8: 25.
4. Yue-Chun L, Gu XH, Li-Sha G, Zhou DP, Xing C, Guo XL, et al. Vagus nerve plays a pivotal role in CD4+ T cell differentiation during CVB3-induced murine acute myocarditis. Virulence. 2021; 12: 360-76.
